# Burden and Risk Factors of Cardiovascular Diseases in Asia, 1990–2021: An Analysis for GBD 2021

**DOI:** 10.1016/j.jacasi.2025.08.024

**Published:** 2025-11-06

**Authors:** Yuxin Zhang, Qiuju Deng, Piaopiao Hu, Qinghui Zeng, Bingxiao Li, Jing Liu

**Affiliations:** Center for Clinical and Epidemiological Research, Beijing An Zhen Hospital, Capital Medical University, Beijing Institute of Heart, Lung and Blood Vessel Diseases, Beijing, China

**Keywords:** cardiovascular disease, Global Burden of Disease, risk factor

## Abstract

**Background:**

Despite ongoing prevention efforts, the burden of cardiovascular disease (CVD) continues to rise with rapidly aging population and lifestyle changes. Comprehensive reports on CVD burden for Asia remain limited.

**Objectives:**

The aims of this study were to quantify the CVD burden in Asia from 1990 to 2021 and to investigate associated risk factors and their temporal rank changes.

**Methods:**

Data from the Global Burden of Disease (GBD) 2021 study were used to estimate the prevalence, disability-adjusted life years (DALYs), and mortality of CVD from 1990 to 2021 across 6 Asian regions, age groups, and sex. Twenty-seven behavioral, environmental, and metabolic risk factors were analyzed.

**Results:**

In 2021, Asia accounted for 270.4 million DALYs and 11.8 million deaths of CVD, representing 61.3% of global CVD mortality. The age-standardized DALYs rate (ASDR) in Asia exceeded the global average by 8.3%. The ASDR was highest in Central Asia but lowest in the high-income Asia Pacific region among the 6 Asian regions. From 1990 to 2021, ASDR and age-standardized mortality rate declined by 27.5% and 25.4%, respectively, whereas age-standardized prevalence increased by 71.7%. Ischemic heart disease and stroke collectively contributed to more than 80% of CVD deaths. High systolic blood pressure persisted as the leading risk factor, while particulate matter pollution emerged as a leading contributor to CVD burden in Asia.

**Conclusions:**

Despite declines in ASDR and age-standardized mortality rate, the absolute CVD burden in Asia has increased sharply. Regional disparities in CVD burden and risk factors profiles highlight the necessity for targeted interventions.

Cardiovascular disease (CVD) is the leading cause of deaths worldwide. Estimated CVD deaths were 18.6 million in 2019, constituting approximately one-third of all global deaths.^1^, ^2^, ^3^, ^4^, ^5^ Despite multinational and multipronged cardiovascular preventive strategies, the global burden of CVD has continued to rise.^3^^,^^6^ In 2019, there were more than 500 million prevalent cases of CVD globally, almost double compared with 1990.^6^^,^^7^ CVD has significant impacts not only on individuals but also on caregivers, families, communities, and society in general, with a tremendous economic burden on health systems in terms of direct (eg, hospitalizations, physician visits, medications) and indirect (eg, loss of productivity because of short- or long-term disability) costs associated with morbidity.^8^, ^9^, ^10^ In Europe and the United States, health care costs related to CVD exceeded €282 billion and $400 billion, respectively, with costs projected to reach $1.3 trillion annually in the United States alone by 2050.^8^^,^^11^

Although CVD was traditionally considered a “Western” disease or a disease of affluence, this is no longer the case.^12^^,^^13^ Compared with Western countries, most Asian countries, except for Japan, South Korea, Singapore, and Thailand, have higher age-adjusted mortality from CVD.^14^^,^^15^ The CVD epidemic in Asia contributed to 60% of total CVD deaths worldwide as of 2019.^1^^,^^14^ Moreover, the CVD burden in Asia was forecasted to continue its upward trend in the years ahead, affected by the rapidly growing aging population and changing lifestyles. On the basis of data from the Institute for Health Metrics and Evaluation and the Global Burden of Disease (GBD) 2019 study, between 2025 and 2050, crude cardiovascular mortality is projected to rise by 91% in Asia, despite a 23% decrease in the age-standardized mortality rate (ASMR).^16^

Asia also faces many challenges in CVD prevention and treatment, with the largest population and diverse ethnicities, cultures, socioeconomic status, and health care systems. Given that CVD are highly preventable, to allow evidence-based interventions and strengthen public efforts to eventually reduce the burden of CVD in Asia, accurate and regularly updated data on CVD prevalence, death, disability, and risk factors are of crucial importance.^17^ Although there is cumulative research analyzing and reporting trends and patterns of CVD burden from 1990 to 2021 since the release of GBD 2021, an expansion and upgrade of GBD 2019 using advanced statistical models to improve data quality and dependability, there has been a lack of analysis of the most recent data on CVD specific to Asia.^4^^,^^18^

Although CVD comprise various subtypes (eg, stroke, atrial fibrillation or flutter), previous research has focused only on selected subtypes (eg, ischemic heart disease [IHD], stroke) or investigated the burden of CVD in selected Asian regions (eg, South Asia).^18^, ^19^, ^20^ By far, a comprehensive profile of CVD burden in Asia has yet to be presented. Hence, we aimed to: 1) quantify CVD prevalence, disability-adjusted life years (DALYs), and deaths, including trends and patterns, from 1990 to 2021; 2) further explore the burden of CVD across 6 Asian regions (Central Asia, the Middle East, Southeast Asia, South Asia, East Asia, and high-income Asia Pacific), countries, age groups, and sex; and 3) investigate risk factors for CVD and their changes in rank over time in Asia.

## Methods

### GBD database

Estimates of data used in this study were retrieved from the GBD database,^21^ coordinated by the Institute for Health Metrics and Evaluation.^22^ The GBD study is a multinational collaborative study, providing iterative and continuous estimates of disease burdens. The GBD database includes multiple data types to compile the widest array of information, including vital registration systems and verbal autopsies for all 288 causes, as well as surveys, census surveillances, cancer registries, police records, open-source databases, and minimally invasive tissue samplings.^18^^,^^23^^,^^24^ To ensure the comparability of data by cause, age, sex, location, and time, the GBD study implemented standardized data processing corrections in all regions, including age-sex splitting, garbage code redistribution, and data completeness assessment. All GBD 2021 estimates are available from the GBD project.^21^

### Cardiovascular prevalence, mortality, and DALYs

Historical estimates of prevalence, mortality, and DALYs were selected and retrieved because these metrics can characterize the fatal and nonfatal disease burden of CVD. A total of 12 CVD causes were examined, which included IHD, stroke, hypertensive heart disease, rheumatic heart disease (RHD), cardiomyopathy and myocarditis, atrial fibrillation and flutter, other cardiovascular and circulatory disease, aortic aneurysm, nonrheumatic valvular heart disease, endocarditis, lower extremity peripheral arterial disease, and pulmonary artery disease. Individual causes of CVD were identified using standard case definitions.

### Cardiovascular risk factors

Prevalence, DALYs, and deaths attributable to behavioral, environmental, and metabolic cardiovascular risk factors were extracted. The standard case definitions used to identify behavioral (eg, dietary habits, tobacco use, low physical activity), environmental (eg, air pollution, nonoptimal temperatures, other environmental risk factors), and metabolic risk factors (eg, high systolic blood pressure [SBP], high body mass index [BMI], kidney dysfunction). A total of 27 risk factors were included and investigated.

### Statistical analysis

A vital registration system, cause-of-death inference records, and a cause-of-death ensemble model were used to estimate cause-specific mortality for CVD, with cause-of-death ensemble model estimates adjusted using a cause-of-death correction algorithm.^25^ Details on the modeling and estimation are described elsewhere.^26^ To account for uncertainty, 95% uncertainty intervals were calculated for each metric, on the basis of the 2.5th and 97.5th percentiles of 1,000 draw-level estimates, which included posterior uncertainty from fixed effects and the 95% quantile of between-study heterogeneity.^27^

In addition to DALYs and deaths, to ensure standardized comparisons between populations with different age structures over time, age-standardized prevalence, age-standardized DALYs rate (ASDR), and ASMR were reported. Age-standardized rates were calculated using the GBD standard population. To validate the accuracy of the trend analysis, we conducted Joinpoint regression analysis to calculate the average annual percentage change for key variables from 1990 to 2021. All data from the GBD 2021 on CVD prevalence, DALYs, deaths, ASDR, and ASMR from 1990 to 2021 in Asia were analyzed and visualized using R version 4.3.3 (R Foundation for Statistical Computing) and RStudio version 1.4.555 (Posit). This study used freely available data sets from the GBD. As a result, an ethics statement was not applicable.

## Results

### Overview of CVD burden in Asia

In 2021, DALYs of CVD in Asia were 270.4 million years. The ASDR in Asia was 5,476.7 per 100,000 years, 8.3% higher than the global average (5,055.8 per 100,000 years). CVD deaths were 11.8 million in Asia, accounting for 61.3% of global CVD death. The ASMR for CVD in Asia (257.5 per 100,000) was slightly higher than the global average (235.2 per 100,000). The age-standardized prevalence of CVD in Asia was 6,782.6 per 100,000, lower than the global average (7,178.7 per 100,000).

**Figure 1:**
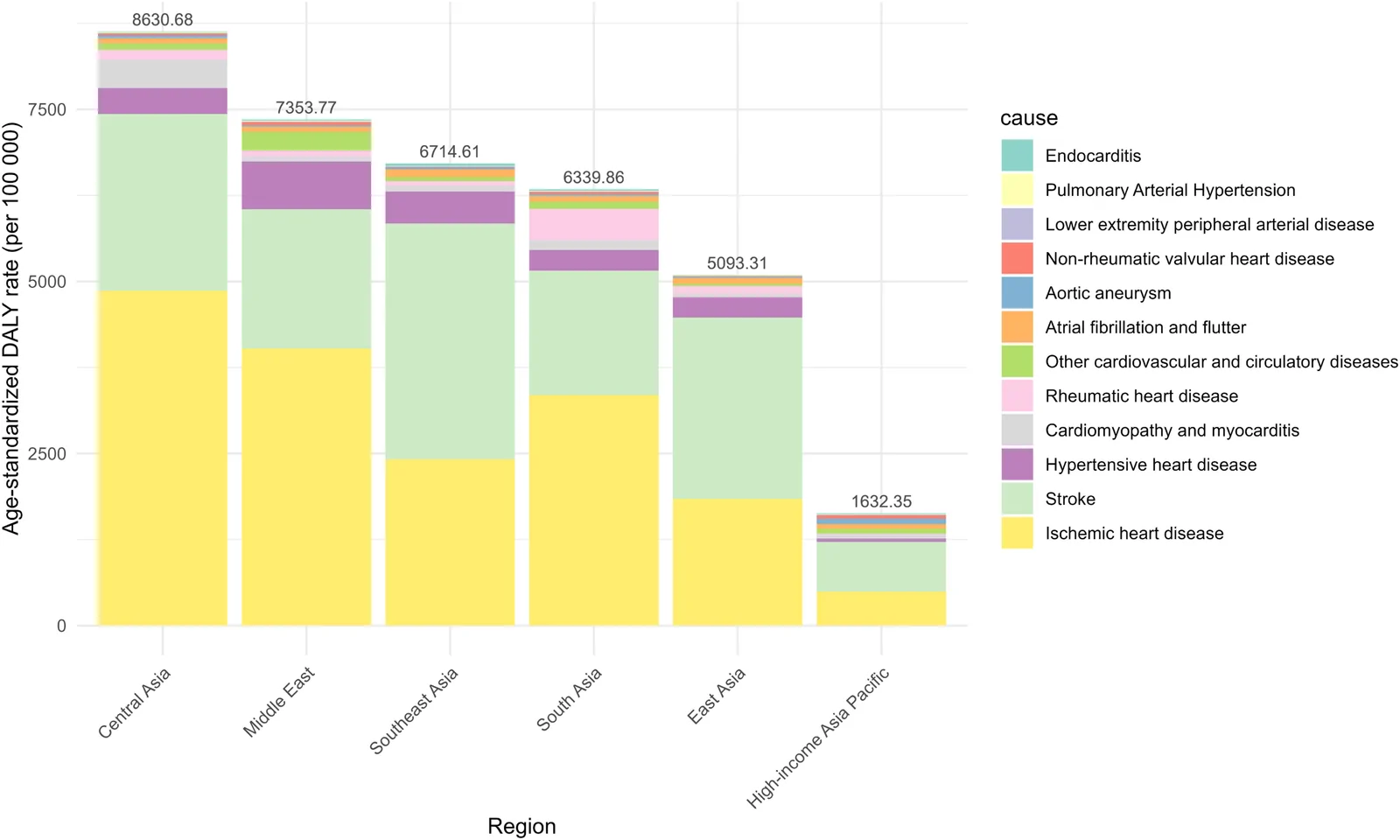
ASDR of Cardiovascular Diseases by Regions in Asia, 2021 Compositional distribution of cardiovascular disease subtype contributions to ASDR across Asian subregions. All data were obtained from the open database of the Global Burden of Disease study in the Global Health Data Exchange. Middle East data are represented by the average data from the Middle East and North Africa. The numbers above the stacked bars represent the total ASDR for each cardiovascular disease subtype in the region. The age-standardized rate is expressed per 100,000 population. Abbreviations as in Figure 1.

**Figure 2:**
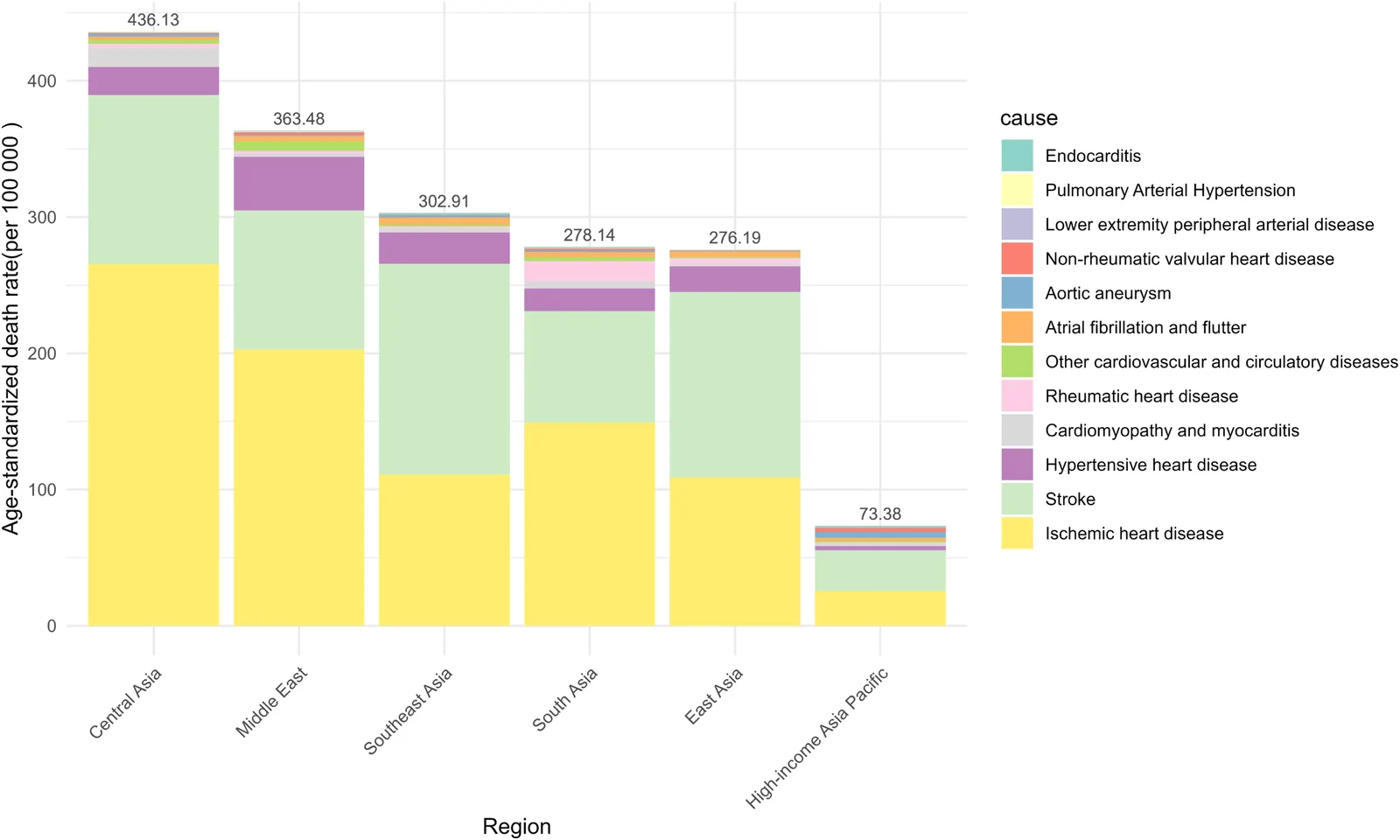
ASMR of Cardiovascular Diseases by Region in Asia, 2021 Compositional distribution of cardiovascular disease subtype contributions to age-standardized mortality rate (ASMR) across Asian subregions. All data were obtained from the open database of the Global Burden of Disease study in the Global Health Data Exchange. Middle East data are represented by the average data from the Middle East and North Africa. The numbers above the stacked bars represent the total ASMR for each cardiovascular disease subtype in the region. The age-standardized rate is expressed per 100,000 population.

**Figure 3:**
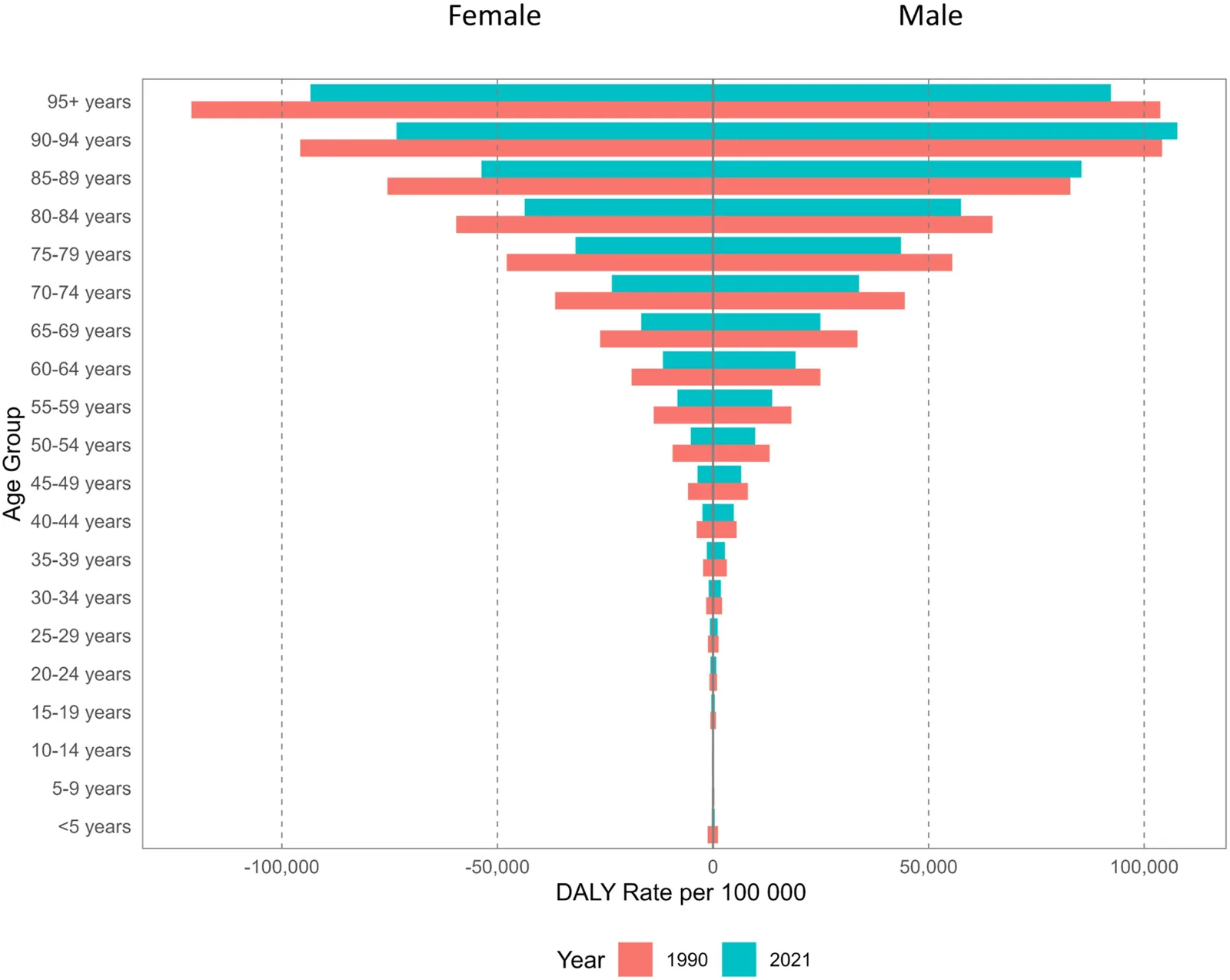
Asia Cardiovascular Disease Age-Standardized DALYs Rate by Age and Sex, 1990 vs 2021 Differences in cardiovascular disease age standardized disability-adjusted life year rate (ASDR) between sexes and across age groups in 1990 vs 2021. All data were obtained from the open database of the Global Burden of Disease study in the Global Health Data Exchange. The age-standardized rate is expressed per 100,000 population. DALYs = disability-adjusted life years.

The ASDR was highest in Central Asia (8,630.7 per 100,000 years) and lowest in the high-income Asia Pacific region (1,632.4 per 100,000 years) (Table 1). In Central Asia, the Middle East, and South Asia, IHD was the main contributor to burden, whereas in East Asia, Southeast Asia, and the high-income Asia-Pacific region, stroke was the main contributor (Figures 1, 2, Supplemental Table 1). In 2021, the ASDR of CVD was 1.5 times higher in men than in women. From 1990 to 2021, although both sexes experienced declines in CVD ASDR, the reduction was greater in women than in men. The ASDR of CVD increased as age increased, with the highest ASDR observed in the ≥95-year age group for women and the 90- to 94 year age group for men in 2021 (Figure 3).

**Table 1:** Age-Standardized Prevalence, ASDR, ASMR of Cardiovascular Disease Regions in Asia

	1990			2021			Change (%)			AAPC		
	ASDR	ASMR	Prevalence	ASDR	ASMR	Prevalence	ASDR	ASMR	Prevalence	ASDR (95% CI)	ASMR (95% CI)	Prevalence (95% CI)
Asia	7,553.3	347.7	6,822.4	5,476.7	259.5	7,301.6	−27.5%	−25.4%	7.0%	−1.1^a^ ( −1.1 to −1.0)	−0.9^a^ (−1.0 to −0.9)	0.2^a^ (0.2−0.2)
Central Asia	10,668.1	512.9	7,749.7	8,630.7	436.1	8,144.0	−19.1%	−15.0%	5.1%	−1.0 ( −1.3 to −0.7)	−0.8 (−1.0 to −0.5)	0.2^a^ (0.2−0.2)
Middle East	1,1421.8	529.2	9,660.1	7,353.8	363.5	9,882.7	−55.3%	−31.3%	2.3%	−1.4^a^ ( −1.09 to −1.0)	−1.2^a^ (−1.3 to −1.1)	0.1^a^ (0.1−0.1)
Southeast Asia	8,110.3	355.5	5,672.0	6,714.6	302.9	5,832.5	−17.2%	−14.8%	2.8%	−0.6^a^ (−0.7 to −0.5)	−0.5^a^ (−0.6 to −0.4)	0.1^a^ (0.1−0.1)
South Asia	7,379.9	302.5	7,070.8	6,339.9	278.1	7,419.9	−14.1%	−8.0%	4.9%	−0.4^a^ (−0.5 to −0.3)	−0.2^a^ (−0.3 to −0.1)	0.2^a^ (0.2−0.2)
East Asia	8,014.8	403.3	2,6047.1	5,093.3	276.2	6,601.7	−36.5%	−31.5%	9.2%	−1.4^a^ ( −1.09 to −1.0)	−1.1^a^ (−1.3 to −0.9)	0.3^a^ (0.3−0.3)
High-income Asia Pacific	3,963.7	204.1	5,704.5	1,632.4	73.4	5,003.5	−58.8%	−64.1%	−12.9%	−2.8^a^ ( −3.0 to −2.7)	−3.2^a^ (−3.4 to −3.0)	−0.5^a^ (−0.6 to −0.4)

The table presents the trends from 1990 to 2021 in age-standardized prevalence, ASDR, and ASMR for cardiovascular diseases across Asia and its subregions, along with corresponding AAPC values derived from Joinpoint regression analysis. Middle East data are represented by the average data from the Middle East and North Africa. All data were obtained from the open database of the Global Burden of Disease study in the Global Health Data Exchange. Change is the difference between the 2021 and 1990 values, divided by the 1990 value.

Abbreviations as in Table 1.

a Results were considered statistically significant (*P* < 0.05). The age-standardized rate is expressed per 100,000 population.

IHD and stroke of CVD ASDRs were 2,340.9 and 2,280.1 per 100,000, respectively, accounting for approximately 80% of CVD deaths, with similar patterns seen in ASMR. Of the 12 subtypes of CVD, IHD had the highest age-standardized prevalence (3,223.6 per 100,000), followed by lower extremity peripheral arterial disease (1,196.4 per 100,000) and stroke (1,137.0 per 100,000), respectively (Table 2). The ASMR of IHD was consistently higher in men compared with women across all age groups (Supplemental Figure 1). The ASMR of stroke was considerably higher in men than in women in the 85- to 89-year and 90- to 94 year age groups, while no obvious differences were observed in age groups 85 years (Supplemental Figure 2).

**Table 2:** Age-Standardized Prevalence, ASDR, and ASMR of Cardiovascular Disease Subtypes in Asia

	1990			2021			Change (%)			AAPC		
	ASDR	ASMR	Prevalence	ASDR	ASMR	Prevalence	ASDR	ASMR	Prevalence	ASDR (95% CI)	ASMR (95% CI)	Prevalence (95% CI)
IHD	3,687.5	117.0	2,884.2	2,340.9	113.3	3,223.6	−36.5	−3.1	11.8	0.0 (−0.1 to 0.0)	0.1^a^ (0.0-0.2)	0.4^a^ (0.4-0.4)
Stroke	2,476.0	172.8	1,165.0	2,280.1	108.2	1,137.0	−7.9	−37.4	−2.4	−1.7^a^ (−1.8 to −1.6)	−1.7^a^ (−1.8 to −1.6)	−0.1^a^ (−0.2 to −0.1)
HHD	515.0	28.1	155.0	300.4	16.7	150.8	−41.7	−40.5	−2.7	−1.6^a^ (−1.8 to −1.3)	−1.5^a^ (−1.8 to −1.3)	−0.3^a^ (−0.4 to −0.1)
RHD	501.9	16.4	610.1	217.2	6.6	609.4	−56.7	−59.8	−0.1	−2.7^a^ (−2.8 to −2.7)	−3.0^a^ (−3.0 to −2.9)	0.1^a^ (0.1-0.2)
Cardiomyopathy and myocarditis	110.9	4.0	33.0	92.4	3.3	41.9	−16.7	−17.7	27.1	−0.5^a^ (−0.6 to −0.4)	−0.4^a^ (−0.5 to −0.3)	1.0^a^ (0.9-1.0)
Atrial fibrillation and flutter	85.1	3.6	507.6	88.0	3.8	529.0	3.4	5.5	4.2	0.1 (0.0-0.1)	0.0 (−0.1 to 0.1)	0.2^a^ (0.1-0.2)
Other cardiovascular and circulatory diseases	83.3	2.1	429.4	74.0	1.7	74.0	−11.2	−20.7	−82.8	−0.3^a^ (−0.3 to −0.2)	−0.5^a^ (−0.6 to −0.5)	1.0^a^ (0.9-1.0)
Aortic aneurysm	19.7	1.0	NA	25.7	1.4	NA	30.7	38.6	NA	0.9^a^ (0.8-1.0)	1.2^a^ (1.1-1.3)	NA
VHD	26.1	1.4	170.0	21.8	1.2	189.5	−16.7	−11.4	11.5	−0.3^a^ (−0.5 to −0.2)	0.0 (−0.3 to 0.2)	0.5^a^ (0.4-0.5)
Endocarditis	24.7	0.8	2.1	18.3	0.7	3.4	−25.7	−15.4	62.4	−1.1^a^ (−1.2 to −1.0)	−0.7^a^ (−0.9 to −0.6)	1.6^a^ (1.5-1.6)
Lower extremity peripheral artery disease	9.8	0.2	1,208.7	9.2	0.2	1,196.4	−6.2	4.4	−1.0	−0.2^a^ (−0.3 to −0.2)	0.4^a^ (0.2-0.5)	−0.1^a^ (−0.1 to −0.1)
PAH	13.4	0.4	2.0	8.7	0.3	2.1	−34.7	−22.5	2.0	−1.0^a^ (−1.2 to −0.9)	−0.4^a^ (−0.6 to −0.1)	0.1^a^ (0.1-0.2)

The table presents changes in age-standardized prevalence, ASDR, and ASMR of different cardiovascular disease subtypes in Asia from 1990 to 2021, along with AAPC results from Joinpoint regression analysis. Change is the difference between the 2021 and 1990 values, divided by the 1990 value. All data were obtained from the open database of the Global Burden of Disease study in the Global Health Data Exchange.

AAPC = average annual percentage change; ASDR = age-standardized disability-adjusted life year rate; ASMR = age-standardized mortality rate; HHD = hypertensive heart disease; IHD = ischemic heart disease; NA = not available; PAH = pulmonary arterial hypertension; RHD = rheumatic heart disease; VHD = nonrheumatic valvular heart disease.

a Results were considered statistically significant (*P* < 0.05). The age-standardized rate is expressed per 100,000 population.

### CVD burden from 1990 to 2021 in Asia

Total DALYs of CVD in Asia in 2021 increased by 74.5% compared with that in 1990 (average annual percentage change = 0.6; *t* = 0.6; *P* < 0.001), while the ASDR in Asia decreased by 27.5% (average annual percentage change = −1.1; *t* = −53.8; *P* < 0.001). CVD deaths in Asia increased by 106.1%, doubling from a 57.5% increase globally. The ASMR of CVD in Asia decreased by 25.4% (Table 1). ASDR in Asia decreased in 2021 compared with those in 1990 across all age groups and among both women and men, except for a small increase in ASDR among men aged 90 to 94 years (Figure 3). Age-standardized prevalence increased by 71.7% from 1990 to 2021, considerably higher than a global increase of 42.9%.

Both ASDR and ASMR of CVD decreased from 1990 to 2021 in all 6 Asian regions. The high-income Asia Pacific region had the highest reduction in ASDR (58.8%) in 2021, followed by the Middle East (55.3%). The high-income Asia Pacific region also experienced a 64.0% reduction in ASMR in 2021 compared with 1990. Age-standardized prevalence increased from 1990 to 2021 in all Asian regions (2.3%-9.2%) except the high-income Asia Pacific region (a reduction of 12.9%) (Table 1).

The ASDRs of all 12 subtypes of CVD decreased, ranging from 6.2 to 56.7%, except atrial fibrillation and flutter and aortic aneurysm, with increases of 3.4 and 30.7% in 2021, respectively. The ASMRs of atrial fibrillation and flutter, aortic aneurysm, and lower extremity peripheral arterial disease increased in 2021, whereas the ASMRs of other subtypes of CVD decreased, where RHD had the highest reduction in ASMR (59.8%) (Table 2).

The age-standardized prevalence of IHD, cardiomyopathy and myocarditis, atrial fibrillation and flutter, nonrheumatic valvular heart disease, endocarditis, and pulmonary arterial hypertension increased in 2021 in comparison with 1990, of which endocarditis had the highest increase (62.4%) in age-standardized prevalence (Table 2).

### CVD risk factors in Asia

**Figure 4:**
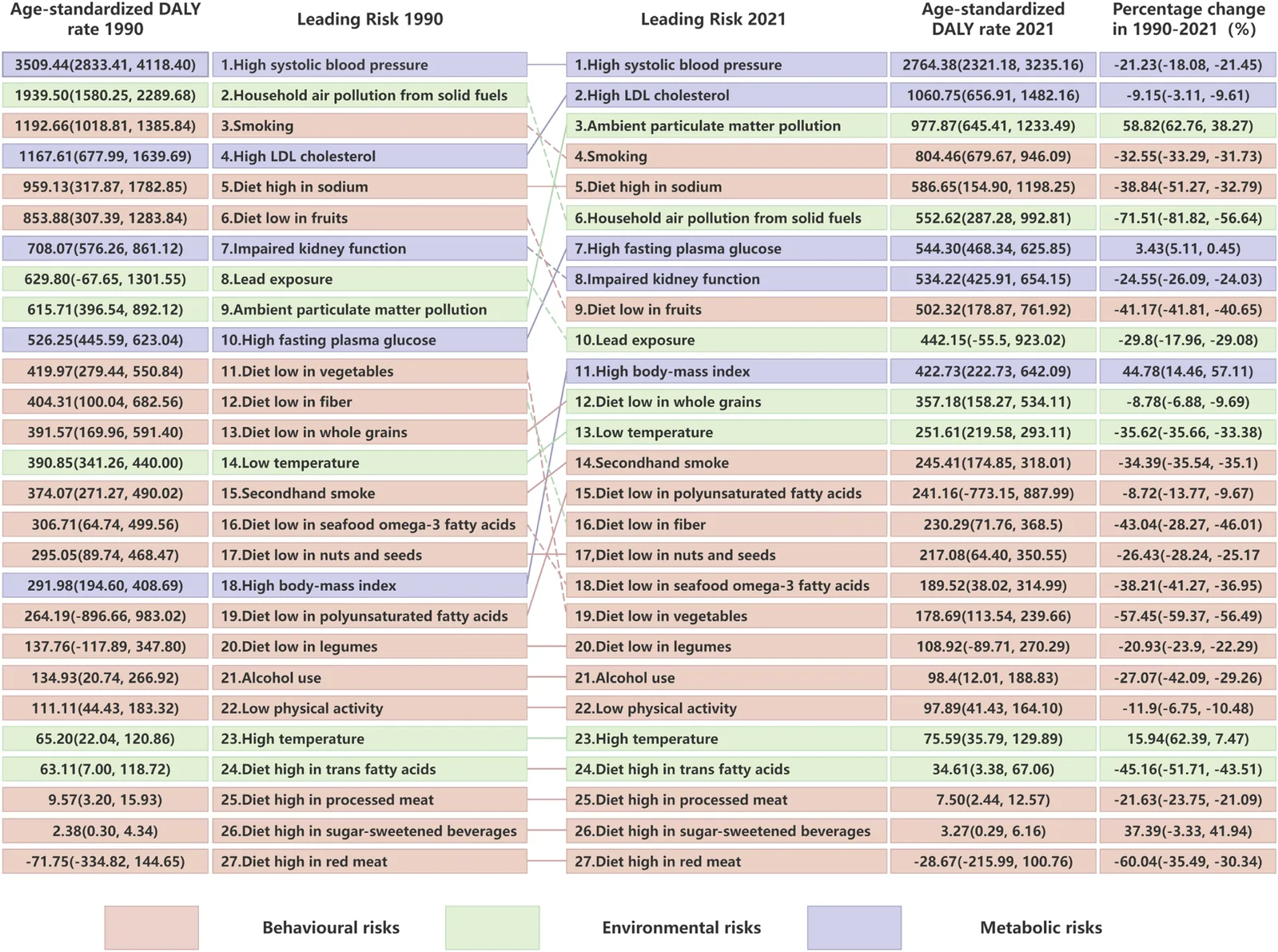
Ranking of 27 Attributable Risk Factors of Cardiovascular Disease in Asia Temporal shifts in ranking and percentage changes of attributable risk factors for cardiovascular diseases across Asia between 1990 and 2021. All data were obtained from the open database of the Global Burden of Disease study in the Global Health Data Exchange. The age-standardized rate is expressed per 100,000 population. DALYs = disability-adjusted life years.

In 1990, the leading risk factors of CVD were high SBP, household air pollution from solid fuels, smoking, high low-density lipoprotein (LDL) cholesterol, and diet high in sodium, all of which have dropped substantially (9.2%-71.5%) in terms of ASDR in 2021. Despite high SBP remaining the leading risk factor of ASDR, household air pollution and smoking, which ranked 2nd and 3rd in 1990, dropped to 6th and 4th in 2021. Notable shifts included the large increase in ASDR (58.8% and 44.8%) and rank for ambient particulate matter pollution and high BMI, respectively, in which ambient particulate matter pollution ranked 3rd in 2021, while it ranked 9th in 1990, and high BMI jumped to 11th from 18th (Figure 4). Smoking was the second attributable CVD ASDR risk factor in men but ranked 19th in women. Alcohol use also ranked higher in men. Notably, household air pollution from solid fuels ranked 4th in women and 8th in men, while secondhand smoke ranked 12th and 17th, both showing greater impact on women (Supplemental Figure 3 and 4). From 1990 to 2021, men in Asia exhibited substantial increases in the rank of CVD ASDR attributable to ambient particulate matter pollution, high fasting plasma glucose, and high BMI but marked declines for household air pollution from solid fuels. Among women, ambient particulate matter pollution, high fasting plasma glucose, and high BMI similarly rose, while diets low in vegetables and smoking decreased.

Overall, the ranks of metabolic risk factors attribute to ASDR moved up in Asia in 2021, except that high SBP remained at the top of all risk factors, and impaired kidney function dropped from seventh to eighth. Ranks of behavioral risk factors, including smoking, diet low in fruit, diet low in vegetables, diet low in fiber, and diet low in seafood omega-3 fatty acids, dropped at varying degrees. Environmental risk factors, including lead exposure, dropped in rank in 2021 (Figure 4). Similar rankings of CVD risk factors were observed in terms of ASMR (Supplemental Figure 5), with slight differences that ambient particulate matter pollution and high LDL cholesterol ranked second and third in 2021, respectively, while high fasting plasma glucose ranked 5th, taking over diet high in sodium.

### CVD risk factors across Asian countries and regions

**Figure 5:**
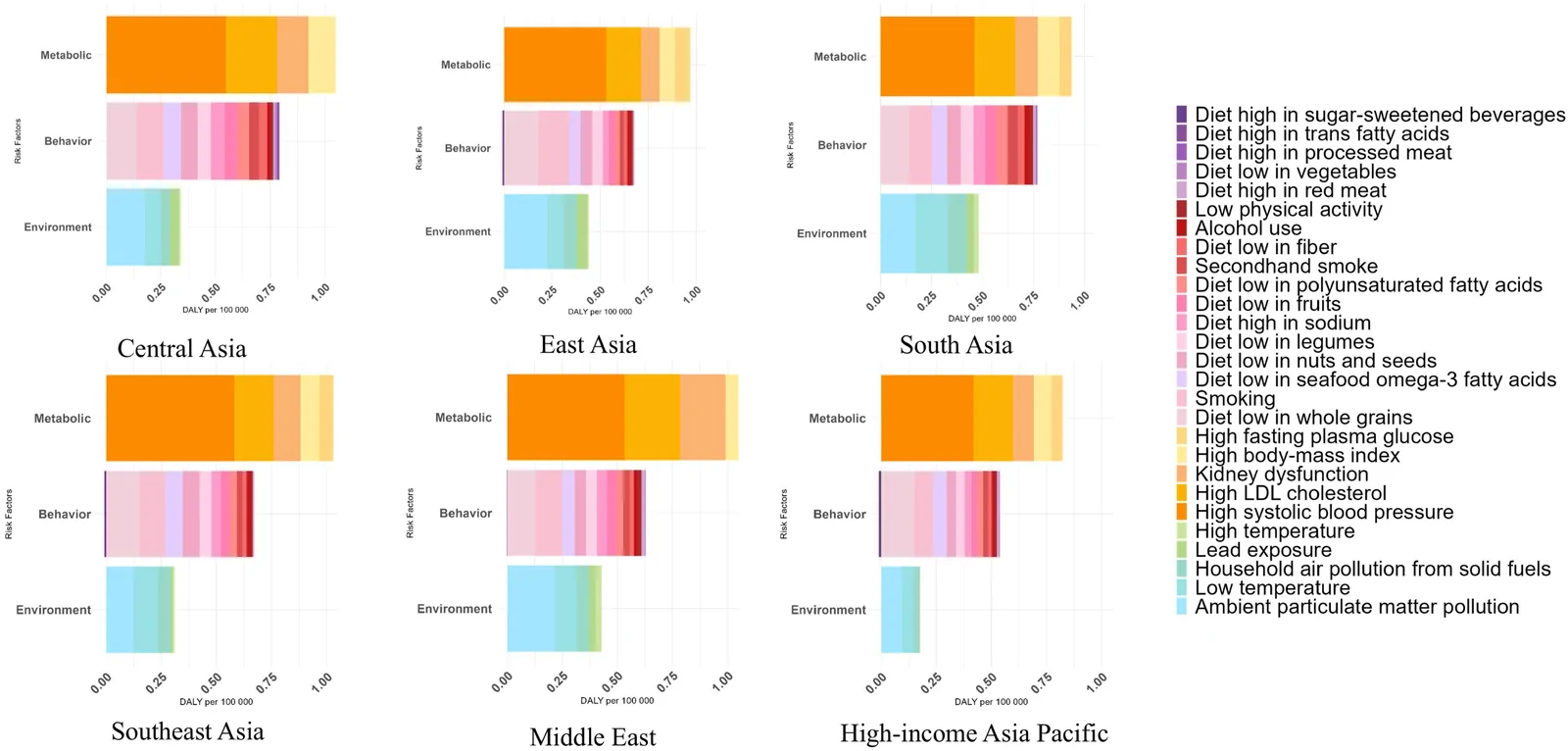
Major Attributable Risk Factors of Cardiovascular Disease Age-Standardized DALYs Rate in Asian Regions, 2021 Distribution of environmental, metabolic, and behavioral risk factors contributing to cardiovascular disease age-standardized disability-adjusted life years (DALYs) rate across different regions. All data were obtained from the open database of the Global Burden of Disease study in the Global Health Data Exchange. Middle East data are represented by the average data from the Middle East and North Africa. The age-standardized rate is expressed per 100,000 population. LDL = low-density lipoprotein.

**Figure 6:**
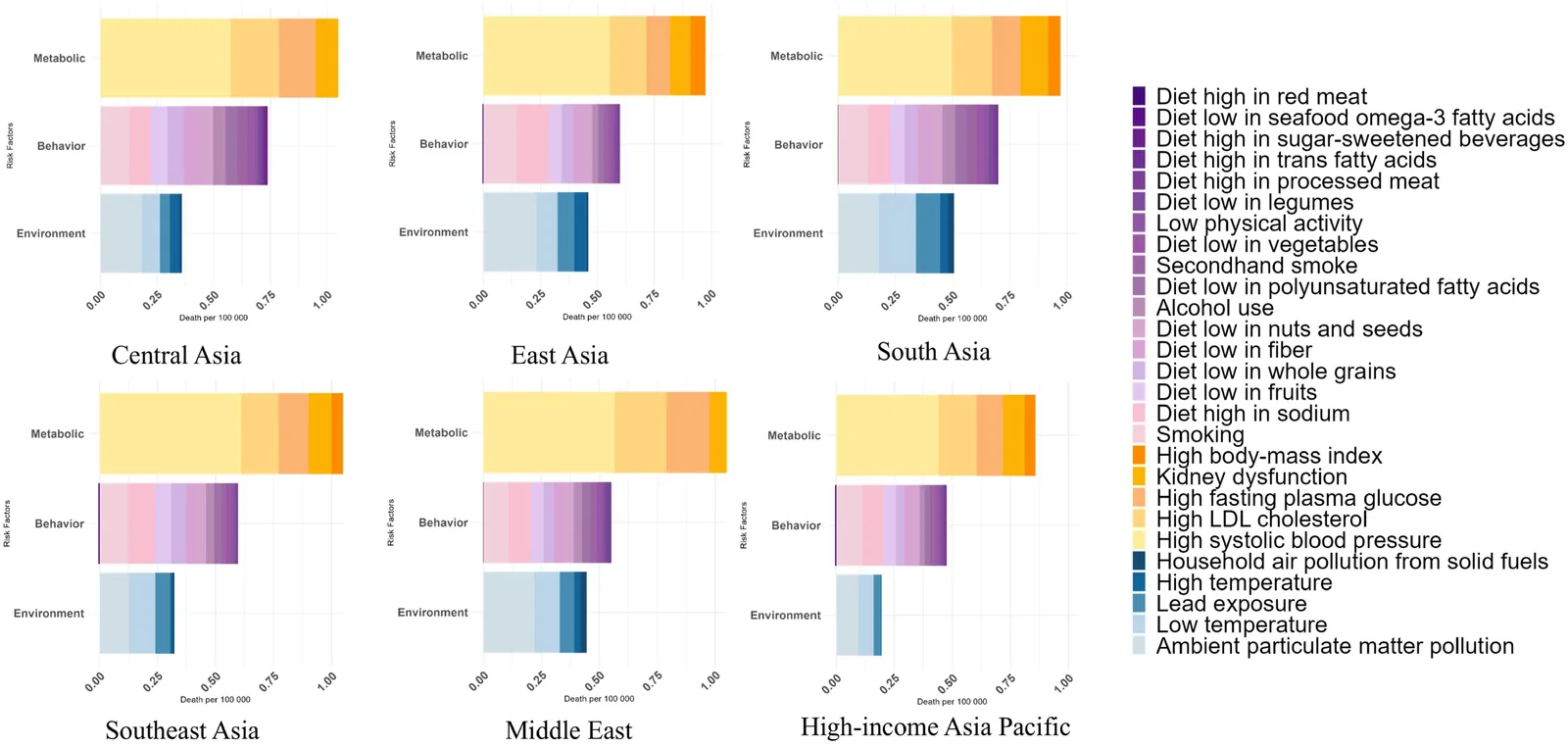
Major Attributable Risk Factors of Cardiovascular Disease ASMR in Asian Regions, 2021 Distribution of environmental, metabolic, and behavioral risk factors contributing to cardiovascular disease age-standardized mortality rate across different regions. All data were obtained from the open database of the Global Burden of Disease study in the Global Health Data Exchange. Middle East data are represented by the average data from the Middle East and North Africa. The age-standardized rate is expressed per 100,000 population. LDL = low-density lipoprotein.

In 2021, ambient particulate matter pollution, high SBP, and high LDL cholesterol ranked among the top 5 attributable risk factors for ASDR of CVD across all Asian subregions. In the attribution of CVD ASDR across Asian subregions, household air pollution from solid fuels and diet low in fruits ranked as the third and fifth leading risk factors, respectively, in South Asia, while smoking fell outside the top 5 in both Central Asia and the Middle East. Kidney dysfunction emerged as the fourth major risk factor in Central Asia and the fifth in Southeast Asia. Concurrently, high BMI placed fifth in Central Asia and fourth in the Middle East, with high fasting plasma glucose ranking fifth in the Middle East and fourth in the high-income Asia Pacific region. The top 5 leading risk factors for CVD ASDR in East Asia align with the overall Asian pattern (Supplemental Table 2). The distribution of ASDR and ASMR for CVD burden attributable to different risk factors across Asian regions in 2021 is detailed in Figures 5 and 6.

Changes in leading risk factors from 1990 to 2021 vary across Asian regions. The change of high BMI ranked first in South Asia and the high-income Asia Pacific region. The change of diet high in sugar-sweetened beverages, alcohol use, and high fasting plasma were leading risk factors in East Asia, Southeast Asia, and Middle East, respectively. The change of high temperature was the top risk factor in Central Asia. Ambient particulate matter pollution remained among the leading risk factors for each Asian region (Supplemental Figure 6).

DALYs attributed to ambient particulate matter pollution increased considerably in Vietnam, Bhutan, Mongolia, India, and Timor-Leste in 2021. DALYs attributable to high fasting plasma glucose increased in nearly one-half of Asian countries, in which Uzbekistan experienced the largest increase, followed by Indonesia and Timor-Leste. DALYs attributable to high SBP increased from 1990 to 2021 in Pakistan, Indonesia, Uzbekistan, Timor-Leste, and Turkmenistan, while substantial reductions were observed in South Korea and Singapore. Significant reductions in DALYs attributed to high LDL cholesterol were observed in Singapore, Qatar, and South Korea. DALYs attributed to smoking declined in most Asian countries except Indonesia, Timor-Leste, Uzbekistan, and North Korea (Supplemental Figure 7).

## Discussion

Our study presents a comprehensive analysis of the burden of CVD in Asia, using data from the GBD 2021 database. The findings reveal significant regional disparities in CVD prevalence, disability, and mortality, highlighting the evolving challenges Asia faces in terms of both the increasing burden of CVD and changing risk factors. In brief, the burden of CVD continued to increase in Asia (Central Illustration), where CVD deaths in Asia accounted for more than 61.3% of global CVD-related deaths in 2021, highlighting the urgent need for targeted interventions in the Asian region. Our findings also revealed that although the ASMR of CVD decreased across most Asian regions since 1990, the total number of CVD-related deaths increased dramatically, particularly in South Asia and the Middle East.

**Central Illustration:**
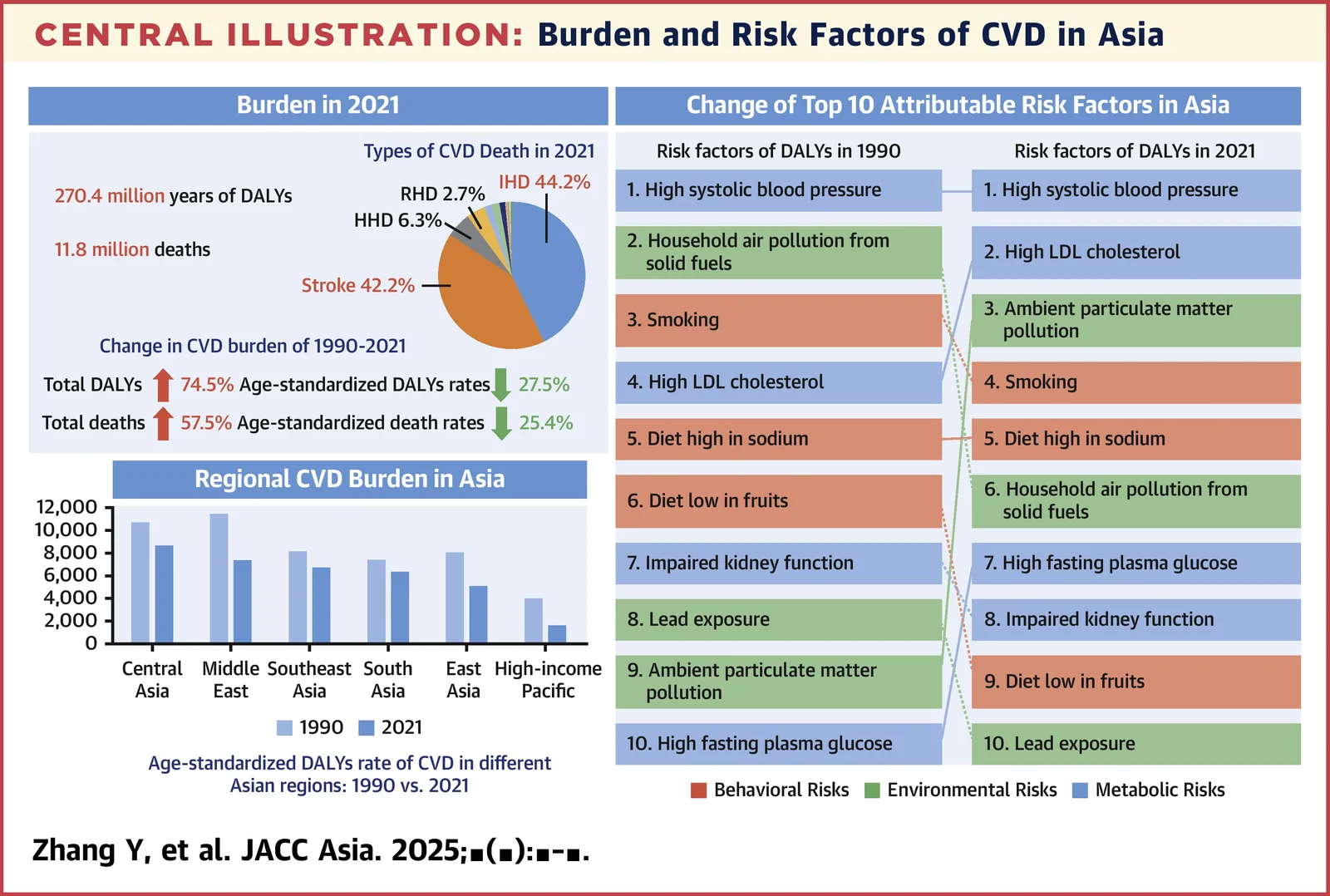
Burden and Risk Factors of CVD in Asia The data used for all analyses were derived from the Global Burden of Disease study 2021. The percentages under “Types of CVD in 2021” represent the proportional prevalence of different cardiovascular disease (CVD) subtypes. From 1990 to 2021, the number of deaths and disability-adjusted life years (DALYs) of CVD increased in Asia, while the age-standardized mortality rate and age-standardized DALYs rate (ASDR) of CVD decreased. Ischemic heart disease (IHD) and stroke were the dominant contributors to DALYs. The high-income Asia Pacific region had the lowest CVD burden, while Central Asia had the highest ASDR in the region. In 2021, high systolic blood pressure was the leading risk factor for CVD in Asia. HHD = hypertensive heart disease; RHD = rheumatic heart disease.

Regional differences were observed in Asia. Central Asia had the highest age-standardized CVD burden, with IHD being the dominant contributor to both DALYs and mortality. This may be attributed to high salt intake, alcohol use, and smoking, which elevate the risk for IHD.^28^^,^^29^ In contrast, the high-income Asia Pacific region (eg, Japan) had a relatively lower CVD burden,^30^ likely due to better health care infrastructure and publicly available preventive measures. Although RHD has been eliminated in high-income countries, it remains a significant public health challenge in South Asia. This is mainly due to poverty, limited health care access, and low health awareness.^31^^,^^32^ These findings highlight the need for region-specific strategies to address the unique challenges posed by CVD in different regions of Asia. Although the high-income Asia Pacific region had seen reductions in both age-standardized prevalence and ASMR, other regions, especially Southeast Asia and South Asia, witnessed substantial increases.

Our findings showed that ASMR in the high-income Asia Pacific and Middle East regions declined substantially. These reductions may be attributed to improvements in health care access, early detection and diagnosis, and treatment of CVD, as well as targeted prevention efforts. However, despite these successes, the overall trends of increasing CVD-related DALYs and deaths across much of the region suggested that more needed to be done to address the underlying determinants of CVD, including socioeconomic inequality, urbanization, and the increasing burden of chronic conditions such as diabetes and high blood pressure.

The age-standardized prevalence of CVD increased by more than 70% from 1990 to 2021 in Asia, much higher than the global increase of 42.9%. This alarming trend reflected both demographic changes, such as an increasing aging population and lifestyle changes, particularly in low- and middle-income Asian countries.^33^ Changes in lifestyle (eg, diet, smoking) may be driving this upward trend in prevalence.^34^^,^^35^ Moreover, risk factors such as high blood pressure, high BMI, and ambient particulate matter pollution emerged as key risk factors for CVD in Asia in 2021.^36^^,^^37^

Although high SBP remains the leading risk factor for CVD in Asia, ambient particulate matter pollution and metabolic risk factors such as high LDL cholesterol have also become top risk factors in 2021. Globally, approximately 8.5 million deaths are associated with SBP exceeding 115 mm Hg, with 88% of these deaths occurring in low- and middle-income countries.^38^ The rise of CVD burden attributed to ambient particulate matter pollution is closely linked to rapid industrialization, widespread use of fossil fuels, increased transportation, and construction activities,^39^ highlighting the pressing need for environmental policies to mitigate air pollution in Asia. Evidence confirms that air quality policies such as China’s Action Plan for Prevention and Control of Air Pollution reduced CVD age-standardized mortality attributable to particulate matter with an aerodynamic diameter ≤2.5 μm by 8.03% within 5 years,^40^ demonstrating that sustained regulatory action can lower the regional risk ranking of ambient pollution within a single policy cycle. Similar strategies^41^ could accelerate reductions in CVD burden in South Asia,^37^^,^^42^^,^^43^ where particulate matter with an aerodynamic diameter ≤2.5 μm remains a leading risk factor.

The geographic variation in the leading risk factors for CVD was also noteworthy. In South Asia, household air pollution from solid fuels is the third leading factor contributing to CVD DALYs. This is due largely to the widespread use of solid fuels such as cow dung and wood, which significantly elevate particulate matter levels, even in households primarily using kerosene, as seen in India.^42^ The regional differences highlighted the need for tailored approaches related to CVD prevention and management in Asia, with consideration of various dietary patterns and cultural practices.

In addition, socioeconomic disparities play a critical role in the heterogeneous distribution of risk factors across Asian regions. High-income regions such as Japan tend to allocate substantial resources toward comprehensive health care infrastructure, nationwide preventive strategies, and rigorous workplace wellness programs that effectively mitigate leading risks.^44^ In contrast, low-income regions such as South Asia face multifaceted challenges from economic constraints. Poverty drives increased reliance on solid fuel use while simultaneously contributing to reduced fruit consumption.^42^ Concurrently, inadequate financial resources limit the development and implementation of effective primary health care services and pollution control strategies.^45^ These economic disparities interact with dietary and cultural differences, giving rise to geographic risk disparities.

A major strength of this study is the comprehensive integration of CVD data across diverse Asian subregions (Central Asia, South Asia, Southeast Asia, and the Middle East), providing unique insights into regional disease patterns and risk factor distributions that can directly inform targeted prevention strategies.

### Study limitations

The data estimates in the GBD study are obtained through a diverse range of data collection methodologies and sources, such as population censuses, household surveys, civil registration systems, vital statistics records, and disease notification mechanisms. This diversity may contribute to data heterogeneity and potentially influence both the completeness and overall quality of the data set. The generalizability of the GBD database may be limited because the same statistical models are uniformly applied across all regions, while data availability constraints force reliance on partial national or subnational data to represent entire geographic areas.

In addition, although age-standardized rates enable comparable CVD burden assessments across regions, the method assumes similar age-outcome relationships across different populations. Given geographic disparities in health care access and early-life environmental exposures, these standardized rates may overestimate or underestimate major outcomes.

## Conclusions

CVD in Asia is a major public health issue and a growing health challenge. Our findings emphasize the need for region-specific strategies to tackle it. Public health policies should focus on addressing the rising prevalence of metabolic risk factors, improving air quality, and promoting healthier lifestyles to mitigate the burden of CVD in Asia. Eventually, continued surveillance and research are essential to monitor the changing trends and patterns of CVD burden and their risk factors in diverse and rapidly changing Asian regions.

## Funding Support and Author Disclosures

This work was partially supported by Capital’s Funds for Health Improvement and Research (2024-1G-3014) and Beijing Hospitals Authority’s Ascent Plan. The authors have reported that they have no relationships relevant to the contents of this paper to disclose.
