# Supplementary material for: Burden and Risk Factors of Cardiovascular Diseases in Asia, 1990–2021: An Analysis for GBD 2021
Source: JACC Asia. 2025 Nov 6;6(8):1750–61. doi: 10.1016/j.jacasi.2025.08.024 (PMC13491087; doi:10.1016/j.jacasi.2025.08.024)
Supplement: Supplementary file 1 [file mmc1.docx]

**SUPPLEMENTAL MATERIAL**

Contents

[Supplemental Figure 1 Asia ischemic heart disease mortality rate per 100,000 by age and sex in 1990 vs. 2021	2](#_Toc210143421)

[Supplemental Figure 2 Asia stroke mortality rate per 100,000 by age and sex in 1990 vs. 2021	3](#_Toc210143422)

[Supplemental Table 1 CVD Percentage Distribution of DALY by region in Asia（2021）	4](#_Toc210143423)

[Supplemental Figure 3 Rank and percent change of leading top 15 risk factors of cardiovascular disease ASDR in Asia in male, 1990 and 2021.	6](#_Toc210143424)

[Supplemental Figure 4 Rank and percent change of leading top 15 risk factors of cardiovascular disease ASDR in Asia in female, 1990 and 2021.	7](#_Toc210143425)

[Supplemental Figure 5 Rank and percent change of leading top 15 risk factors of cardiovascular disease ASMR in Asia, 1990 and 2021.	8](#_Toc210143426)

[Supplemental Table 2 Top 5 risk factors attributed to CVD ASDR in Asian Regions, 2021	9](#_Toc210143427)

[Supplemental Figure 6 The percentage change in DALY attributed to all risk factors for cardiovascular disease in Asia regions	10](#_Toc210143428)

[Supplemental Figure 7 Changes in CVD DALY from Top 5 risk factors in Asian countries (1990-2021)	11](#_Toc210143429)

## Supplemental Figure 1 Asia ischemic heart disease mortality rate per 100,000 by age and sex in 1990 vs. 2021

## Supplemental Figure 2 Asia stroke mortality rate per 100,000 by age and sex in 1990 vs. 2021

## Supplemental Table 1 CVD Percentage Distribution of DALY by region in Asia（2021）

| Cause | Central Asia | East Asia | High-income Asia Pacific | Middle East | South Asia | Southeast Asia |
| --- | --- | --- | --- | --- | --- | --- |
| Ischemic heart disease | 56.36% | 36.12% | 30.20% | 54.71% | 52.86% | 35.97% |
| Stroke | 29.73% | 51.74% | 44.36% | 27.56% | 28.47% | 51.01% |
| Hypertensive heart disease | 4.36% | 5.75% | 2.89% | 9.41% | 4.76% | 6.92% |
| Rheumatic heart disease | 1.65% | 2.18% | 0.80% | 1.18% | 7.15% | 0.98% |
| Cardiomyopathy and myocarditis | 4.79% | 1.04% | 3.54% | 0.99% | 2.24% | 1.22% |
| Atrial fibrillation and flutter | 0.87% | 1.76% | 4.23% | 1.03% | 1.39% | 1.72% |
| Other cardiovascular and circulatory diseases | 1.10% | 0.61% | 4.33% | 3.70% | 1.66% | 0.89% |
| Aortic aneurysm | 0.49% | 0.26% | 4.88% | 0.27% | 0.39% | 0.39% |
| Non-rheumatic valvular heart disease | 0.28% | 0.11% | 2.75% | 0.57% | 0.42% | 0.13% |
| Endocarditis | 0.07% | 0.09% | 0.94% | 0.24% | 0.38% | 0.55% |
| Lower extremity peripheral arterial disease | 0.14% | 0.16% | 0.57% | 0.13% | 0.14% | 0.15% |
| Pulmonary Arterial Hypertension | 0.15% | 0.17% | 0.51% | 0.20% | 0.13% | 0.07% |

## Supplemental Figure 3 Rank and percent change of leading top 15 risk factors of cardiovascular disease ASDR in Asia in male, 1990 and 2021.

Legend: DALY = disability-adjusted life year. ASDR = age-standardized DALY rate. Change is the difference between the 2021 and 1990 values, divided by the 1990 value.

## Supplemental Figure 4 Rank and percent change of leading top 15 risk factors of cardiovascular disease ASDR in Asia in female, 1990 and 2021.

Legend: DALY = disability-adjusted life year. ASDR = age-standardized DALY rate. Change is the difference between the 2021 and 1990 values, divided by the 1990 value.

## Supplemental Figure 5 Rank and percent change of leading top 15 risk factors of cardiovascular disease ASMR in Asia, 1990 and 2021.

Legend: ASMR = age-standardized mortality rate. Change is the difference between the 2021 and 1990 values, divided by the 1990 value.

## Supplemental Table 2 Top 5 risk factors attributed to CVD ASDR in Asian Regions, 2021

| Rank of risk factors | Asia | East Asia | Central Asia | Middle East | South Asia | Southeast Asia | High-income Asia Pacific |
| --- | --- | --- | --- | --- | --- | --- | --- |
| 1 | High systolic blood pressure | High systolic blood pressure | High systolic blood pressure | High systolic blood pressure | High systolic blood pressure | High systolic blood pressure | High systolic blood pressure |
| 2 | High LDL cholesterol | Ambient particulate matter pollution | High LDL cholesterol | High LDL cholesterol | High LDL cholesterol | High LDL cholesterol | High LDL cholesterol |
| 3 | Ambient particulate matter pollution | High LDL cholesterol | Ambient particulate matter pollution | Ambient particulate matter pollution | Household air pollution from solid fuels | Smoking | Smoking |
| 4 | Smoking | Smoking | Kidney dysfunction | High body-mass index | Ambient particulate matter pollution | Ambient particulate matter pollution | High fasting plasma glucose |
| 5 | Diet high in sodium | Diet high in sodium | High body-mass index | High fasting plasma glucose | Diet low in fruits | Kidney dysfunction | Ambient particulate matter pollution |

Legend: ASDR = age-standardized DALY rate. LDL = low density lipoprotein. Middle East data is represented by the average data of the Middle East and North Africa.

## Supplemental Figure 6 The percentage change in DALY attributed to all risk factors for cardiovascular disease in Asia regions

Legends: DALYs=disability-adjusted life years

## Supplemental Figure 7 Changes in CVD DALY from Top 5 risk factors in Asian countries (1990-2021)

Legends: DALY=disability-adjusted life year. Change is the difference between the 2021 and 1990 values, divided by the 1990 value.
